# *Gentiana scabra* Restrains Hepatic Pro-Inflammatory Macrophages to Ameliorate Non-Alcoholic Fatty Liver Disease

**DOI:** 10.3389/fphar.2021.816032

**Published:** 2022-01-18

**Authors:** Yiyuan Zheng, Dan Fang, Chaoyuan Huang, Lina Zhao, Liming Gan, Youlan Chen, Fengbin Liu

**Affiliations:** ^1^ Department of Gastroenterology, The First Affiliated Hospital of Guangzhou University of Chinese Medicine, Guangzhou, China; ^2^ Lingnan Medical Research Center, Guangzhou University of Chinese Medicine, Guangzhou, China; ^3^ Department of Oncology, Shanghai University of Traditional Chinese Medicine Longhua Hospital, Shanghai, China; ^4^ The First Clinical Medical School, Guangzhou University of Chinese Medicine, Guangzhou, China; ^5^ Department of Gastroenterology, Zhongshan Hospital of Traditional Chinese Medicine, Zhongshan, China; ^6^ Baiyun Hospital of The First Affiliated Hospital of Guangzhou University of Chinese Medicine, Guangzhou, China

**Keywords:** *Gentiana scabra*, non-alcoholic fatty liver disease, anti-inflammation, macrophages, experimental evaluation

## Abstract

Non-alcoholic fatty liver disease (NAFLD) has become a progressive metabolic disease that is emerging as a global epidemic. Considering that the complex pathogenesis has not been fully elucidated, barely specific pharmacological therapy is recommended in current guidelines. *Gentiana scabra* (GS) is a commonly used herb in Tibetan medicine, which has received much attention in recent years due to its diverse pharmacological properties, including anti-inflammation, anti-oxidation, and anti-fibrosis. However, the therapeutic mechanisms are still unclear. Our investigation demonstrated a regulatory effect of GS on pro-inflammatory macrophages, which was extensively investigated in NAFLD that revealed intimate participation in the disease evolution, and the non-canonical IKK family member TANK-binding kinase 1 (TBK1) was involved in this process. Plasmid vectors for shTBK1 and amlexanox (AML), an inhibitor of TBK1, were used in this study to verify the mechanisms of TBK1 both *in vitro* and *in vivo*, while a co-culture system for hepatocytes and BMDMs was constructed to confirm the critical role of macrophages for inflammatory cascade. The results revealed that metabolic burden up-regulated the phosphorylation of TBK1, resulting in activation of NF-κB signaling pathway, and consequently caused an elevated expression of MCP1 to induce the macrophage recruitment and accelerate the inflammatory cascade. In contrast, GS could inhibit the TBK1 phosphorylation and the MCP1 expression to restrain the recruitment of pro-inflammatory macrophages, so as to provide curative effects on metabolic dysfunction and inflammation. Considering that GS is non-toxic and can be used as a kind of tea for long-term drinking, we propose it may be an effective option for the prevention and treatment of NAFLD, which deserves further exploration and application, and may provide new insights to improve the current standardized intervention strategy.

## Introduction

Non-alcoholic fatty liver disease (NAFLD) is a clinicopathological syndrome closely related to obesity, insulin resistance, and many other metabolic diseases. Thus it has been defined as metabolic-associated fatty liver disease (MAFLD) in the latest international expert consensus statement (Eslam et al., 2020). Epidemiological investigation shows that the global prevalence of NAFLD has exceeded 25%, of which about 30% can progress to non-alcoholic steatohepatitis (NASH), a more severe and progressive form taken up core position in the pathogenesis of NAFLD (Fan et al., 2017; Younossi, 2018). The evidence suggests that NAFLD has become the leading cause of chronic liver diseases, seriously endangers human health, and causes a substantial economic burden on society (Younossi et al., 2018). However, there is barely specific pharmaceutical therapy recommended for the treatment of NAFLD in current guidelines, hence research on new therapeutic strategies is urgently needed (Aller et al., 2018).

*Gentiana scabra* (GS), a commonly used herb in Tibetan medicine, is the flower of Gentian and possesses multiple pharmacological effects, such as anti-bacterial, anti-viral, anti-tumor, etc.; besides these, many other therapeutic functions have taken more place in recent years, including anti-inflammation, anti-oxidation, and anti-fibrosis (Li et al., 2008; Geng et al., 2010; Khobrakova et al., 2017). Researches have demonstrated that GS could inhibit the activation of hepatic stellate cells resulting in an alleviation of fibrosis formation, as well as might regulate lipid peroxidation to improve hepatic dysfunction, and our study revealed that its extract significantly ameliorated liver steatosis and inflammation in high-fat-fed mice (Cao et al., 2014). Therefore, considering that GS is non-toxic and can be used as a kind of tea for long-term drinking, we propose it is an effective option for the prevention and treatment of NAFLD, and there is thus an imperative need to clarify the pharmacological mechanism to promote its clinical application.

The pathogenesis of NAFLD is multifactorial and has not yet been precisely clarified. However, the multiple-hit theory is widely accepted to a certain extent, whereby parallel hits from the gut and adipose tissues promote hepatic fat accumulation and inflammation, leading to persistent liver injury and repairment, and eventually developing fibrosis (Tilg and Moschen, 2010; Fang et al., 2018). According to this theory, lipids accumulation is a critical event in the early stage of NAFLD, while progression to NASH is fueled by inflammation; herein, innate immunity is a major contributor in which liver-resident macrophages (Kupffer cells) and recruited macrophages are crucial components (Zhang et al., 2014; Krenkel and Tacke, 2017). Evidence shows that hepatic macrophages are inflammatory sensors response to various damage-associated molecular patterns and other signals associated with the increased metabolic burden (Remmerie and Scott, 2018). Specifically, macrophage activation is driven by gut-derived endotoxins, lipid metabolites and molecules released from lipo-apoptotic hepatocytes, while alterations in gut microbiota and defined nutritional components are also significant participants (Hirsova et al., 2016; Alharthi et al., 2020). During this process, liver-resident macrophages differentiate into the pro-inflammatory phenotype and help recruit blood-derived monocytes to aggravate inflammation (Leroux et al., 2012). Notably, both experimental and clinical studies suggest that the pro-inflammatory phenotype of macrophages is closely relative to disease severity, and the recruited monocytes represent a more pro-inflammatory profile compared with liver-resident Kupffer cells, which means pharmacological agents targeting macrophage recruitment can be a promising approach for NAFLD treatment (Morinaga et al., 2015; Kazankov et al., 2019).

Researches have demonstrated that the monocyte flux is primarily mediated by an interaction between monocyte chemotactic protein 1 (MCP1) and its cognate receptor C-C chemokine receptor type 2 (CCR2), which is found highly expressed in monocyte-derived macrophages (Obstfeld et al., 2010). And remarkably, our study found that GS could significantly reverse the increased expressions of MCP1 and CCR2 in liver tissues and effectively improve the macrophage polarization induced by high-fat diets (HFD). Further investigations indicated that the non-canonical IKK family member TANK-binding kinase 1 (TBK1), a bi-directional regulator at the crossroads of inflammation and energy homeostasis, was involved in this mechanism (Zhao et al., 2018). Hence, we proposed and performed experiments to confirm that GS could regulate TBK1, thus attenuating the downstream inflammatory cascade, so as to inhibit the MCP1 expression and the macrophage recruitment.

## Materials and Methods

### Drug Preparation

GS extract used in this study was obtained from Nanjing PuYi Biological Technology Co. Ltd. According to the manufacturer, GS was crushed into coarse powder, added with 12 folds of 50% ethanol, heated and refluxed for extraction. The drug solution was mixed, filtered, and concentrated under reduced pressure. Subsequently, it was dissolved, added to the D101 macroporous adsorption resin column, washed and eluted with 95% ethanol. Finally, the eluent was collected, concentrated, dried and ground into fine powder.

### Liquid Chromatography-Mass Spectrometry Analysis

The qualitative analysis was performed by Beijing Chemdow Co. Ltd. GS extract was diluted in a mobile phase composed of 55% methanol and 45% water. The acquisition was carried out on LCMS-IT/TOF (Shimadzu, Japan), while parameters were set as follows: the column (5020-88027, Shimadzu, Japan) temperature was 30°C, the loading volume was 10 μl, and the flow rate was 1 ml/min. The quasi-molecular ions including (M-H)^−^, (M-H + HCOOH)^−^, (M + H)^+^ and (M + Na)^+^ were estimated as precursor ions, and the ion chromatogram is shown in Supplementary Figure S1.

### Bioinformatic Analysis

The typical components of GS were retrieved manually as their extensive pharmacological activities due to no information recorded in the traditional Chinese medicine system pharmacology (TCMSP, https://old.tcmsp-e.com/) database, a computational platform containing various aspects of herbal information and widely used for systematic pharmacology-based analysis (Ru et al., 2014; Le and Le, 2016). Furthermore, candidate targets for each component were obtained from TCMSP and DrugBank (https://www.drugbank.ca/) database, and the compound-target network was constructed with Cytoscape 3.8.2 software (https://cytoscape.org/) (Shannon et al., 2003). Subsequently, known NAFLD-related targets were collected from Online Mendelian Inheritance in Man database (OMIM, http://www.omim.org/), GeneCards Database (GCD, https://www.genecards.org/), and Therapeutic Target Database (TTD, http://www.omim.org/). Protein-protein interaction (PPI) networks were constructed for GS- and NAFLD-related targets respectively, as well as were intersected by the Cytoscape plugin, Bisogenet (Martin et al., 2010). Finally, putative targets were predicted by degree centrality (DC) value, an index that represented the topological importance of a node in its network which was computed by a plugin named CytoNCA (Tang et al., 2015).

### Animal Model

Male C57BL/6 mice aged 6 weeks were obtained from Guangdong Medical Laboratory Animal Center. A total of 32 mice were reared under standard conditions and randomized equally into four groups after acclimation. HFD (D12492, Research Diets, United States) with 60 kcal% fat was used in this study to construct the NAFLD mice model for 12 weeks, while GS was administered intragastrically at a dose of 100 mg/kg/d as intervention therapy, whereas amlexanox (AML, HY-B0713, MedChemExpress, United States) was given as positive control by oral gavage at a dose of 25 mg/kg/d. All mice were handled humanely and had access to food and water *ad libitum*.

Body weights were recorded weekly, and liver weights were measured at the end of this experiment. Blood samples were drawn from the heart under anesthesia, while livers were excised, and either immediately snap-frozen in liquid nitrogen or fixed in 4% paraformaldehyde (PFA, BL539A, Biosharp, China) for further detections. Intraperitoneal glucose tolerance test (IPGTT) and insulin tolerance test (ITT) were handled as described previously, that glucose levels were measured at time points of 0, 15, 30, 60, 90 and 120 min after intraperitoneal injection of glucose (G8270, Sigma, United States) and insulin (abs42019847, Absin, China) respectively (Zheng et al., 2021). All these experiments were performed under a project license (TCMF1-2019049) approved by the Institutional Animal Care and Use Committee of Guangzhou University of Chinese Medicine.

### Cell Culture

Six-week-old male C57BL/6J mice were used to isolate primary hepatocytes and bone marrow-derived macrophages (BMDMs) as described previously (Chen et al., 2019; Zheng et al., 2021). Mice were anesthetized, fixed, and dissected. Livers were separated and ground with liquid nitrogen. Density gradient centrifugation was conducted to purify hepatocytes, and gelatine (48722, Sigma, United States) was used to promote cell adherence. Meanwhile, the femur and tibia bones were isolated and cut to obtain marrows, which were filtrated through 70 μm cell strainer (352350, BD Biosciences, United States), incubated with red blood cell lysis buffer (R1010, Solarbio, China), and centrifuged to purification. Finally, the isolated bone marrow cells were cultured with IMDM (L0191, Biowest, France) containing 10 ng/ml M-CSF (ab129146, Abcam, United Kingdom) for the co-culture assay. The planking concentrations for hepatocytes and BMDMs were 2 × 10^5^ and 1 × 10^5^ cells/ml, respectively, and the incubation time of co-culture was 24 h.

Palmitic acid (PA, P5585, Sigma, United States) was applied in this research to establish lipotoxic cell model, whereas GS and AML were used for intervention. PA, AML and GS were all dissolved in dimethyl sulfoxide (D8418, Sigma, United States), and subsequently, added to culture medium (containing 0.1% BSA) for 24 h at the final concentrations of 0.3 mM, 1 μM and 0.5 μg/ml, respectively. Plasmid vectors coding for TBK1-specific shRNA named shTBK1 were obtained from Shanghai Obio Technology Co. Ltd., and were transfected into hepatocytes by Lipofectamine 2000 transfection reagent (11668027, Thermo Fisher, United States) according to the relevant instructions. The ratio of plasmid to lipo was 1:2 in working medium, and the transfection time is 48 h.

### Quantitative Real-Time Polymerase Chain Reaction

Total RNA was extracted according to protocols by TRIzol (15596026, Invitrogen, United States), and the concentrations were measured using Thermo Scientific NanoDrop 2000c (Waltham, United States). Subsequently, PrimeScript RT reagent (RR036A, Takara, Japan) and TB Green Premix Ex Taq II (RR420A, Takara, Otsu, Shiga, Japan) were applied to conduct qRT-PCR based on Bio-Rad CFX96 Real-Time PCR System (Hercules, United States). The relative mRNA levels of target genes were calculated by 2−ΔΔCt, and beta-actin was used to normalize the samples. All primer sequences used in this research are listed in Table 1.

**TABLE 1:** List of primer sequences used in this study.

Gene name	Sequence (5′-3′) forward	Sequence (5′-3′) reverse
TNF-α	CCC​TCA​CAC​TCA​GAT​CAT​CTT	GCT​ACG​ACG​TGG​GCT​ACA​G
IL-1β	AGT​TGA​CGG​ACC​CCA​AAA​G	AGC​TGG​ATG​CTC​TCA​TCA​GG
MCP1	TAA​AAA​CCT​GGA​TCG​GAA​CCA	GCA​TTA​GCT​TCA​GAT​TTA​CGG​GT
EMR1	CTG​CAC​CTG​TAA​ACG​AGG​CTT	GCA​GAC​TGA​GTT​AGG​ACC​ACA​A
Beta-actin	CTCTCCCTCACGCCATC	ACGCACGATTTCCCTCTC

### Western Blotting

Western blot analyses were performed as described previously (Zheng et al., 2021). Total proteins were extracted from hepatocytes or liver samples using RIPA lysis buffer (P0013B, Beyotime, China), separated by SDS-PAGE gel electrophoresis, and transferred to polyvinylidene fluoride membranes (ISEQ00010, Millipore, United States). The membranes were blocked with 5% milk (232100, BD Biosciences, United States), and subsequently hybridized overnight with primary antibodies at 4°C. Finally, the secondary antibody (1:10000, 14708, CST, United States) was used to combine chemiluminescent horseradish-peroxidase substrate (WBKLS0500, Millipore, United States), and the membranes were imaged by Bio-Rad ChemiDoc XRS System (Hercules, United States).

Primary antibodies against p-TBK1 (1:2000, 5483), p-NF-κB (1:2000, 3033) and beta-actin (1:2000, 4970) were obtained from Cell Signaling Technology (Beverly, United States), against TBK1 (1:1000, sc-398366) and NF-κB (1:1000, sc-8008) were purchased from Santa Cruz Biotechnology (California, United States), and against CCR2 (1:1000, abs136993) was purchased from Absin (Shanghai, China).

### Histological Staining

Histological staining was performed as described previously (Zheng et al., 2021). Liver tissues were fixed, processed, and embedded into paraffin blocks for hematoxylin and eosin (H&E) staining, as well as into optimal cutting temperature (4583, Sakura, United States) compound for oil red O staining. Liver sections were stained by immunohistochemistry kit (G1215, Servicebio, China) with antibodies against TBK1 (1:100), NF-κB (1:100) and CCR2 (1:200), and the images were acquired on a Leica Dim8 microscope (Weztlar, Germany). The quantitative analyses were calculated by ImageJ 1.8.0 (National Institues of Health, United States), whereas the NAFLD activity score (NAS) was computed by steatosis, intralobular inflammation and hepatocyte ballooning to assess disease severity (Brunt et al., 2011).

### Fluorescent Staining

Pre-treated hepatocytes were fixed with 4% PFA for 15 min, blocked with 3% bovine serum albumin (HY-D0842, MedChemExpress, United States) for 1 h, permeabilized with 0.3% Triton X-100 (T8787, Sigma, United States) for 15 min, and hybridized with antibodies against TBK1 (1:200), NF-κB (1:200) and MCP1 (1:200, abs120679, Absin, China) overnight at 4°C. Secondary antibodies conjugated with 488/594 (1:500, SA00006-2/4, Proteintech, United States) were incubated for 1 h at room temperature, and DAPI (1:1000, C1002, Beyotime, China) was used to detect nuclei. Antibodies against iNOS (1:500, GB11119) and CD11b (1:500, GB11058) for liver sections were purchased from Servicebio (Wuhan, China), and images were obtained on an Olympus BX-50 microscope (Tokyo, Japan).

### Flow Cytometry

Mitochondrial membrane potential assay kit with JC-1 (C2006, Beyotime, China) and reactive oxygen species assay kit (S0033S, Beyotime, China) were conducted for pre-treated hepatocytes according to the instructions as described previously (Zheng et al., 2021). Liver samples were separated, washed, ground, and filtrated to a single-cell suspension. About 1×10^6^ cells were diluted into 100 ul phosphate buffered solution for subsequent staining. Antibodies against F4/80 FITC (123107), CD11b PE (101207), CD206 PE-CY7 (141719), and CD86 APC (105011) were obtained from BioLegend (California, United States), while 5 μl of each antibody (1:20) was added into samples and incubated for 1 h. Ad interim, fixation and permeabilization solution (554714, BD Biosciences, United States) was applied according to its manual. And finally, data acquisition was conducted on NovoCyte D2060R (Agilent, United States), and the analyses were performed by FlowJo v10.8 (BD Biosciences, United States).

### Statistical Analysis

Data were expressed as mean ± SD, and the analyses were performed by GraphPad Prism 7.0 (GraphPad Software, United States). One-way analysis of variance (ANOVA) was conducted for data analyses among groups, and *p* value <0.05 was considered statistically significant.

## Results

### Bioinformatics Analysis Identified Pharmacodynamic Targets of GS

Considering that no reasonable standard has been established for quality detection of GS so far, gentiopicrin is considered as a control in this study as it is the standard sample for Gentian according to Chinese Pharmacopoeia, and reported to be largely contained in GS (Lu et al., 2009; Olennikov et al., 2015). Ion chromatogram displayed that this ingredient was identified in GS extract, which was revealed as a component with a mass of 381 m/z at the peak time of 5.833 min that was estimated as gentiopicrin combined with a sodium ion, and thus the quality standard was established (Supplementary Figure S1).

**FIGURE 1:**
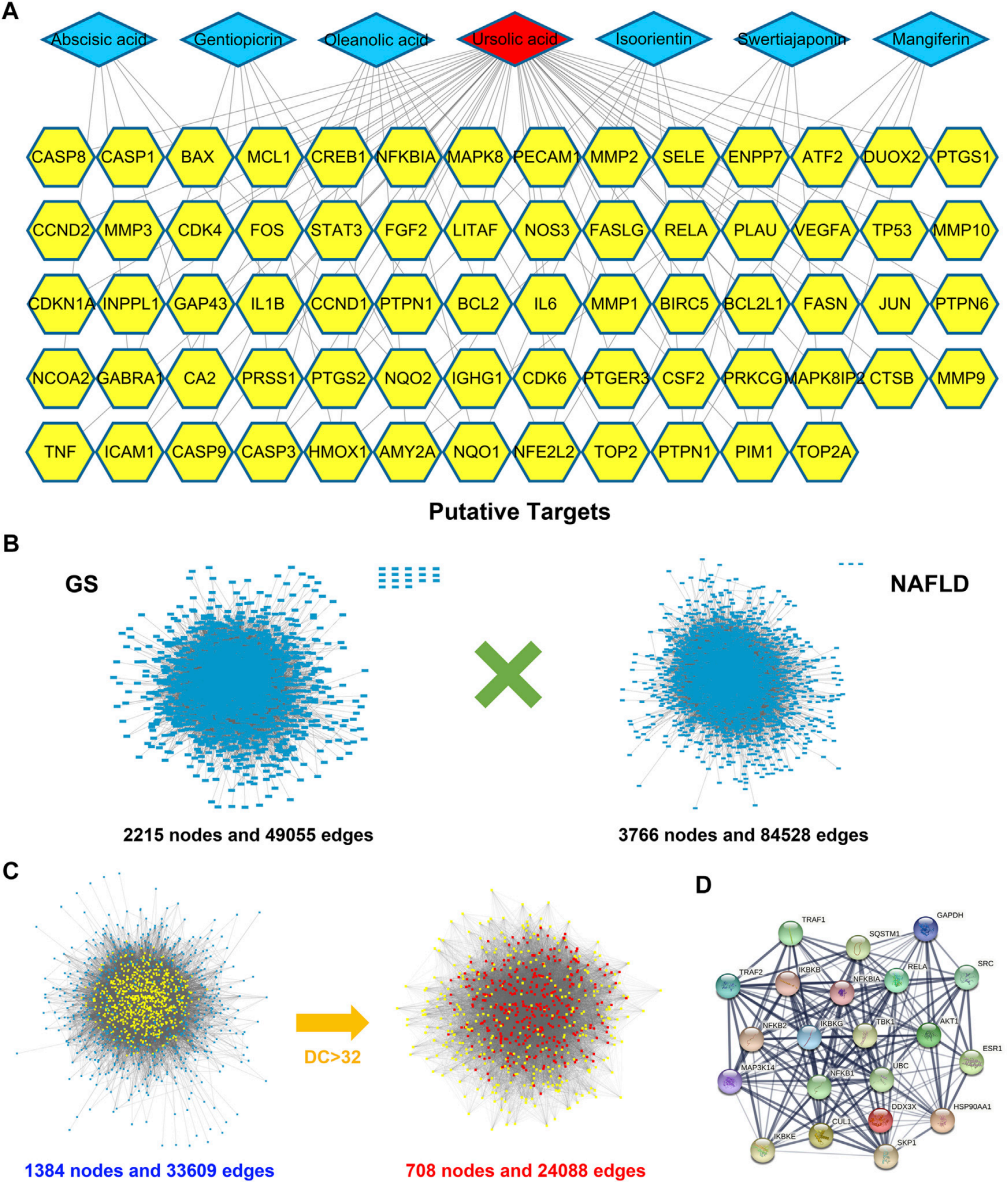
Bioinformatics analysis identified pharmacodynamic targets of GS. **(A)** A compound-target network was constructed for the seven candidate compounds of GS and 76 corresponding targets. **(B)** PPI networks were separately constructed for 76 potential targets of GS and 112 known NAFLD-related targets. **(C)** PPI networks were intersected to identify the putative targets of GS against NAFLD, which were screened by DC value. **(D)** An inflammation-related signaling network including TBK1 was achieved from the intersected network.

Up to now, researches on GS are still rare, resulting in no definite pharmacological ingredients can be found in databases. Therefore, literature was retrieved manually, and seven active components were included in this study for pharmacological analysis, including gentiopicrin, isoorientin, mangiferin, ursolic acid, abscisic acid, oleanolic acid, and swertiajaponin (Supplementary Table S1) (Xu et al., 2008; Lu et al., 2009; Cao et al., 2014). A compound-target network was constructed for the candidate compounds and 76 corresponding targets obtained from TCMSP and DrugBank database, and the result showed that ursolic acid took a considerable weight in the network, suggesting it might be an important bioactive constituent (Figure 1A and Supplementary Table S2). Subsequently, PPI networks were separately constructed for 112 known NAFLD-related targets retrieved from databases (containing 2215 nodes and 49055 edges) and 76 potential targets of GS (containing 3766 nodes and 84528 edges); meanwhile, an intersected network containing 1384 nodes and 33609 edges was used to identify the candidate targets of GS against NAFLD (Figures 1B,C and Supplementary Table S3). Finally, 708 putative targets were achieved under the conditions of DC > 32 (the median value), and an inflammation-related signaling network including TBK1 was found to occupy a large part in the network (Figures 1C,D).

### GS Improved Inflammation and Insulin Resistance in High-Fat-Fed Mice

**FIGURE 2:**
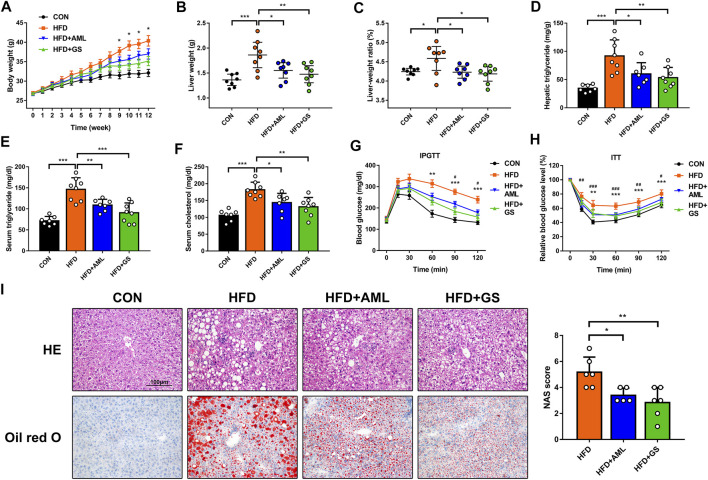
GS improved inflammation and insulin resistance in high-fat-fed mice. **(A)** The body weights were recorded weekly, *n* = 8. *p < 0.05 between the HFD group and the GS group. **(B, C)** The liver weights and liver-to-body weight ratio were measured and calculated at the end of this experiment, *n* = 8. **(D–F)** The hepatic and serum concentrations of triglyceride and cholesterol were detected by ELISA, *n* = 6–8. **(G, H)** IPGTT and ITT, *n* = 7–8. **p < 0.01, ***p < 0.001 between the HFD group and the GS group. ^*#*^ *p < 0.05,* ^##^ *p < 0.01,* ^###^ *p < 0.001* between the HFD group and the AML group. **(I)** HE and oil red O staining, while the NAS score was computed by steatosis, intralobular inflammation and hepatocyte ballooning to assess disease severity, *n* = 6. Results are presented as means ± SD. *p < 0.05, **p < 0.01, ***p < 0.001.

High-fat-fed mice were employed in this study to establish the NAFLD mice model. The difference in body weight between the standard diet group and the HFD group was significant, and administration of GS obviously mitigated the weight gain, whereas AML provided a slight improvement, indicating that GS could alleviate obesity induced by HFD (Figure 2A). Accordingly, liver weight and liver-to-body weight ratio were declined in the GS and AML groups compared with the HFD group, demonstrating that GS might also ameliorate heatic fat accumulation (Figures 2B,C). Biochemical analyses revealed that both GS and AML could abate the elevated triglyceride, cholesterol, alanine aminotransferase and aspartate aminotransferase triggered by HFD, while IPGTT and ITT displayed that they could also provide protective effects against glucose tolerance and insulin resistance, suggesting substantial improvement in metabolic abnormalities (Figures 2D–H and Supplementary Figure S2). Furthermore, HE and oil red O staining were applied, and the results displayed much more severe pathological damages in the HFD group, involving infiltration of numerous neutrophils and accumulation of lipid droplets; in comparison, GS distinctly ameliorated these phenomena that significantly reduced the NAS score, presenting biological functions of anti-inflammation and anti-steatosis (Figure 2I).

### GS Regulated TBK1 to Restrain Inflammatory Cascade

**FIGURE 3:**
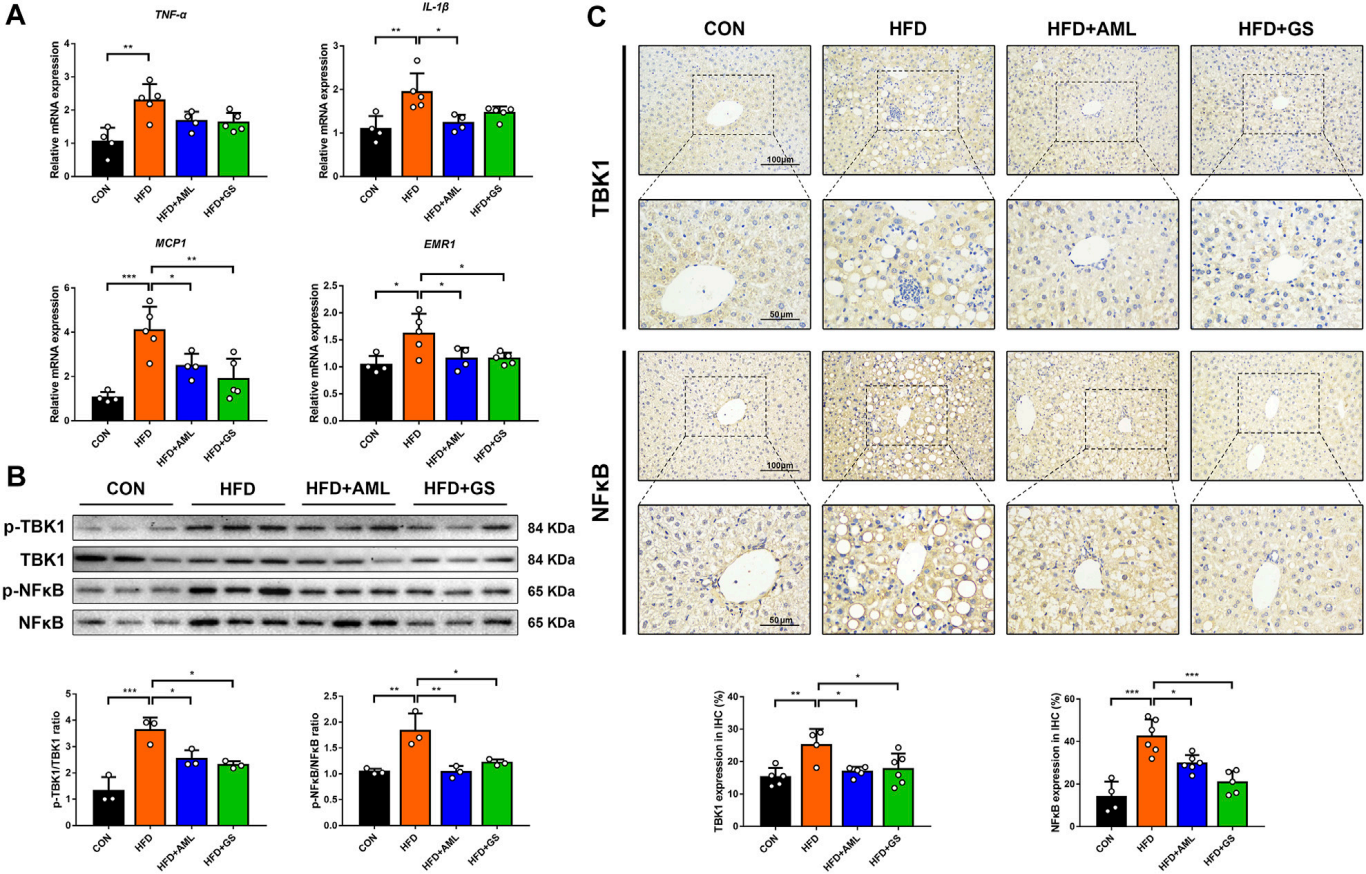
GS regulated TBK1 to restrain inflammatory cascade. **(A)** The relative mRNA expressions in liver tissues were determined by PCR, *n* = 4–5. **(B)** Western blot analyses of p-TBK1, TBK1, p-NF-κB and NF-κB in liver tissues and statistical graphs, *n* = 3. **(C)** Immunohistochemical staining of TBK1 and NF-κB and statistical graphs, *n* = 4–6. Results are presented as means ± SD. *p < 0.05, **p < 0.01, ***p < 0.001.

PCR analyses presented that gene levels of inflammatory indicators of TNF-α and IL-1β were significantly increased in the HFD group, while the MCP1 expression ascended correspondingly as the downstream signal, represented an activation of inflammatory cascade (Figure 3A). The intervention of GS resulted in mild declines in TNF-α and IL-1β as well as a distinct reduction in MCP1, suggesting that pharmacodynamic mechanisms might attribute to partially related signaling pathways. Considering that TBK1 was discovered to be a major target of GS in our previous study, western blot analyses and immunohistochemistry were performed to detect the protein expressions and phosphorylation levels of TBK1 and its downstream indicator, NF-κB. The outcomes showed that the protein and phosphorylation levels of TBK1 and NF-κB were all increased in the HFD group, whereas were obviously suppressed by GS and AML, demonstrating that GS could inhibit this signaling to restrain inflammatory cascade (Figures 3B,C). Meanwhile, GS also strikingly reversed the elevated expression of EMR1, a biomarker of macrophages, indicating that GS might regulate the macrophage recruitment (Figure 3A).

### GS Inhibited Pro-Inflammatory Macrophage Recruitment

**FIGURE 4:**
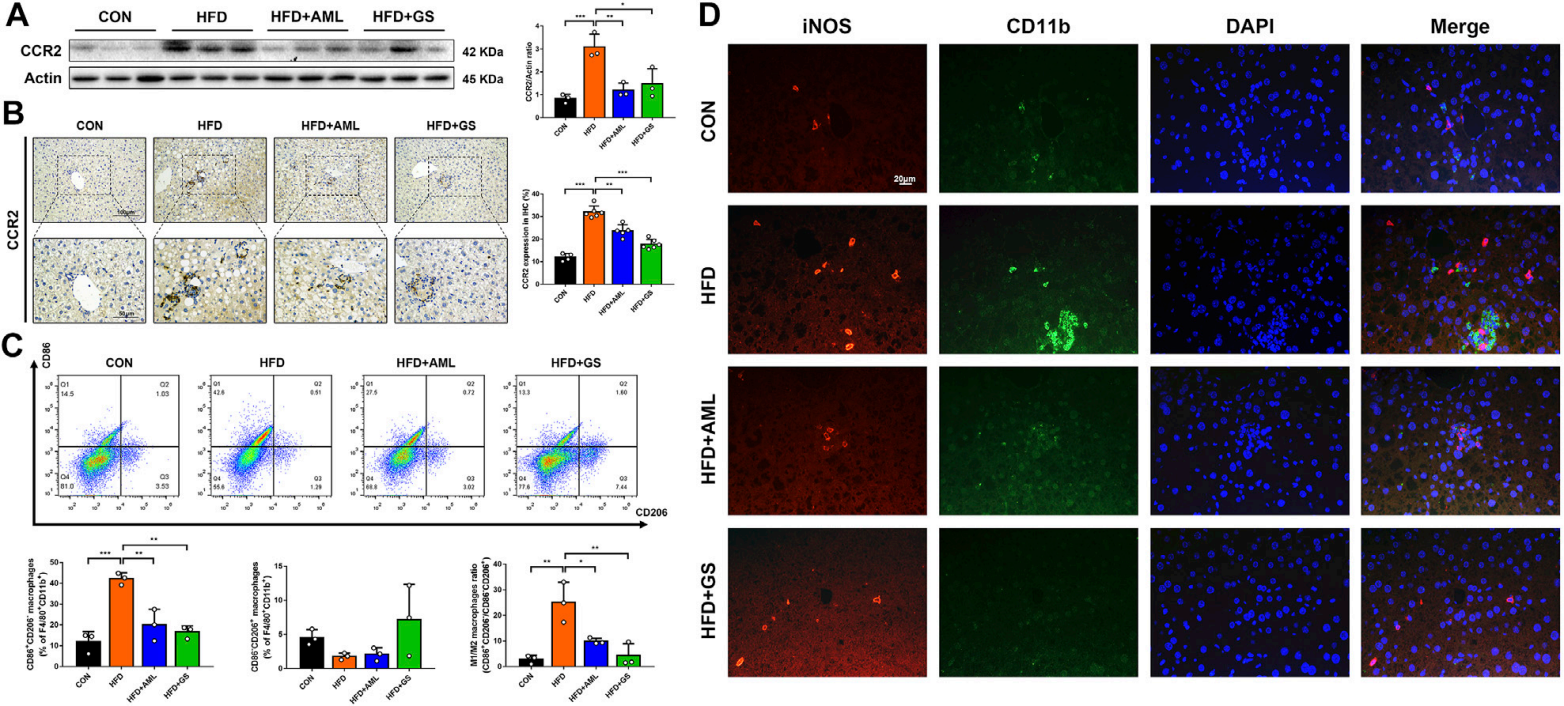
GS inhibited pro-inflammatory macrophage recruitment. **(A)** Western blot analysis of CCR2 in liver tissues and statistical graph, *n* = 3. **(B)** Immunohistochemical staining of CCR and statistical graph, *n* = 4–6. **(C)** The phenotype of macrophages in liver tissues was measured by flow cytometry, *n* = 3. F4/80^+^CD11b^+^ was labeled to represent the macrophages, while CD86^+^CD206^-^ macrophages were recognized as M1 phenotype, and CD86^−^CD206^+^ macrophages were recognized as M2 phenotype. **(D)** Double immuno-fluorescence staining of iNOS and CD11b. Results are presented as means ± SD. *p < 0.05, **p < 0.01, ***p < 0.001.

Western blot analysis revealed that CCR2 was significantly up-regulated in the HFD group, and the immunohistochemistry result displayed a clustered expression, whereas the intervention of GS and AML apparently mitigated these phenomena, suggesting an inhibition of macrophage recruitment (Figures 4A,B). To further explore the regulatory effect of GS on macrophages, the phenotype of macrophages in liver tissues was measured by flow cytometry. A surface marker of macrophages, CD86 was labeled to represent the pro-inflammatory phenotype, namely M1 phenotype; whereas CD206 was signed to identify alternative macrophages, commonly referred to as M2 phenotype (Kazankov et al., 2019). The results indicated an evidently increased ratio of pro-inflammatory macrophages in the HFD group, while both GS and AML decreased the proportion of M1 macrophages by reducing its quantity (Figure 4C). The immunofluorescence staining revealed a conspicuous cluster in the HFD group consisted with previous outcomes, which was represented as co-expressed indicators of CD11b and iNOS, indicating an infiltration of pro-inflammatory macrophages; and in comparison, GS prominently improved this situation, suggesting that GS could restrain pro-inflammatory macrophage recruitment (Figure 4D).

### GS Regulated TBK1 to Improve Oxidative Stress in PA-Stimulated Hepatocytes

**FIGURE 5:**
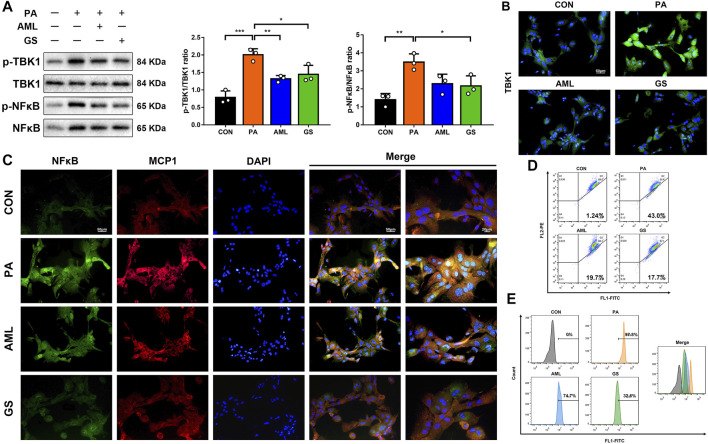
GS regulated TBK1 to improve oxidative stress in PA-stimulated hepatocytes. **(A)** Western blot analyses of p-TBK1, TBK1, p-NF-κB and NF-κB in hepatocytes and statistical graphs, *n* = 3. **(B)** Immuno-fluorescence staining of TBK1. **(C)** Double immuno-fluorescence staining of NF-κB and MCP1. **(D)** The mitochondrial membrane potential assay was detected by JC-1, three samples were collected together for testing, and the experiment was repeated three times. Aggregates with red fluorescence in normal cells were distributed in the second quadrant, while monomers with green fluorescence delivered in the third quadrant represented decreased mitochondrial membrane potential. **(E)** The ROS assay was detected with DCFH-DA, three samples were collected together for testing, and the experiment was repeated three times. Results are presented as means ± SD. *p < 0.05, **p < 0.01, ***p < 0.001.

Primary hepatocytes were isolated and cultured to explore the mechanisms of GS *in vitro*, and PA was used to build a lipotoxic cell model. Western blot analyses showed that the protein levels of p-TBK1 and p-NF-κB were both increased in the PA group, while GS depressed the phosphorylation significantly (Figure 5A). Fluorescence staining also displayed that the expressions of TBK1, NF-κB and MCP1 were all intense in hepatocytes stimulated with PA, whereas were obviously weakened in the GS group, suggesting that GS could regulate this inflammatory signaling pathway to inhibit the MCP1 expression, so as to impair the macrophage recruitment (Figures 5B,C). Considering that the production and accumulation of ROS is a progressive factor for NAFLD to activate oxidative stress, which can injure the function of mitochondria, JC-1 and DCFH-DA were also detected in this research (Rives et al., 2020). The results revealed that aggregates with red fluorescence distributed in the second quadrant were abundant in normal hepatocytes stained with JC-1, whereas was evidently reduced in PA-treated cells; on the contrary, monomers with green fluorescence delivered in the third quadrant were significantly increased in PA-stimulated hepatocytes, demonstrating that mitochondrial membrane potential was reduced and the integrity was damaged (Figure 5D). Furthermore, the ROS content was dramatically ascended in PA-treated cells, and all these phenomena were remarkably alleviated by the intervention of GS, indicating protective effects on mitochondrial function and oxidative stress (Figure 5E).

### TBK1 Promoted NFκB-MCP1 Signaling Pathway

**FIGURE 6:**
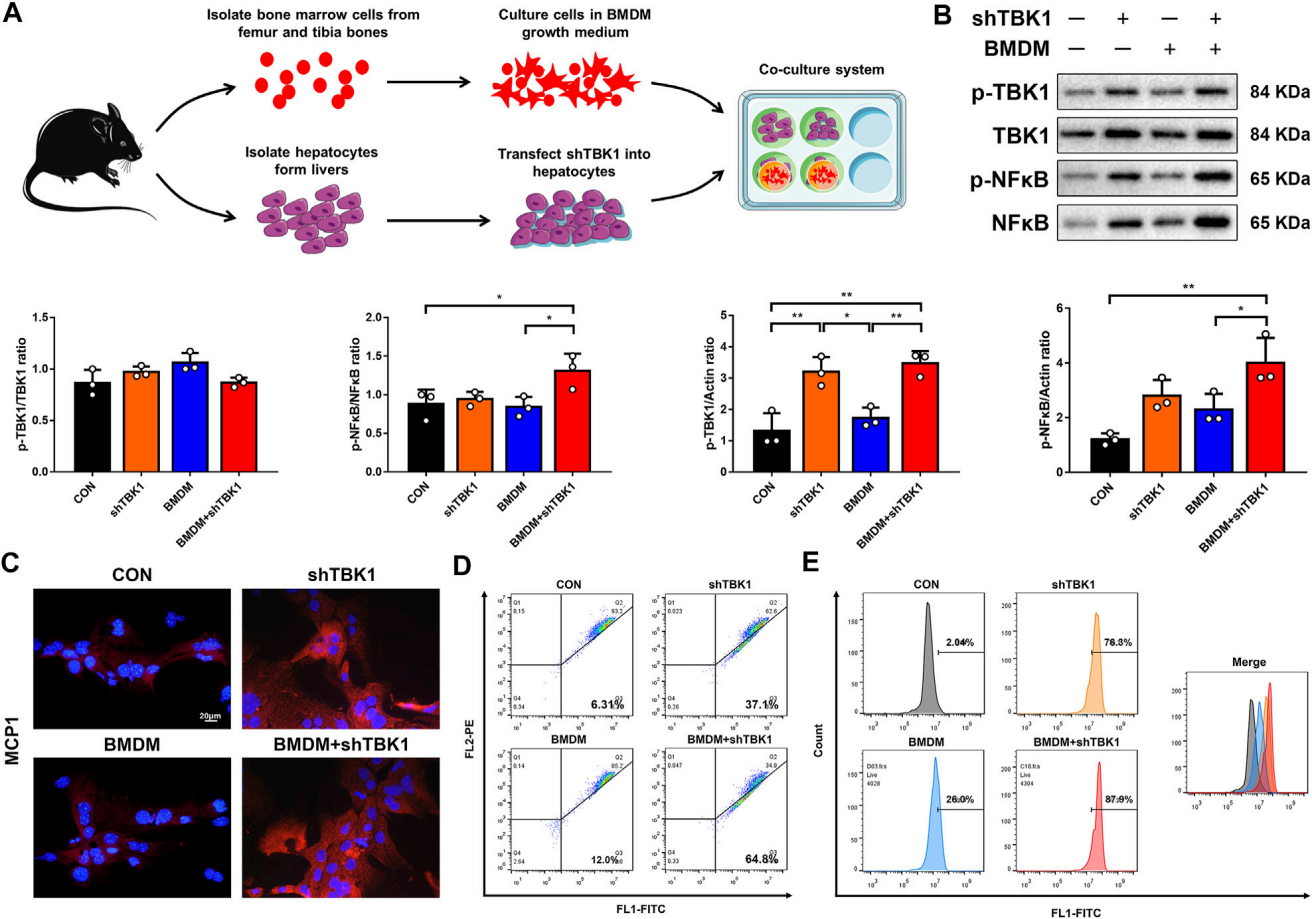
TBK1 promoted NFκB-MCP1 signaling pathway. **(A)** The schematic diagram of co-culture system for hepatocytes and BMDMs. **(B)** Western blot analyses of p-TBK1, TBK1, p-NF-κB and NF-κB in hepatocytes and statistical graphs, *n* = 3. **(C)** Immuno-fluorescence staining of MCP1. **(D)** The mitochondrial membrane potential assay was detected by JC-1, three samples were collected together for testing, and the experiment was repeated three times. **(E)** The ROS assay was detected with DCFH-DA, three samples were collected together for testing, and the experiment was repeated three times. Results are presented as means ± SD. *p < 0.05, **p < 0.01, ***p < 0.001.

To further investigate the exact mechanisms of pro-inflammatory macrophages as well as the TBK1 signaling pathway, a co-culture system for hepatocytes and BMDMs was established; meanwhile, plasmid vectors for shTBK1 were transfected into hepatocytes to induce TBK1-overexpression cell model (Figure 6A). Western blot analyses presented that the protein levels of TBK1 and NF-κB were raised significantly in hepatocytes transfected with shTBK1, while immunofluorescence displayed a simultaneously increased expression of MCP1, suggesting a stable and effective transfection (Figures 6B,C). However, the phosphorylation of NF-κB did not reveal higher ascending until the co-cultured system was added, indicating that macrophages played an essential role in inflammatory cascade. JC-1 and DCFH-DA were detected again, and the results showed that the transfection of TBK1 brought about the production and accumulation of ROS, leading to mitochondrial damage, while the co-cultured macrophages exacerbated these conditions, consisting with our previous founding (Figures 6D,E).

**FIGURE 7:**
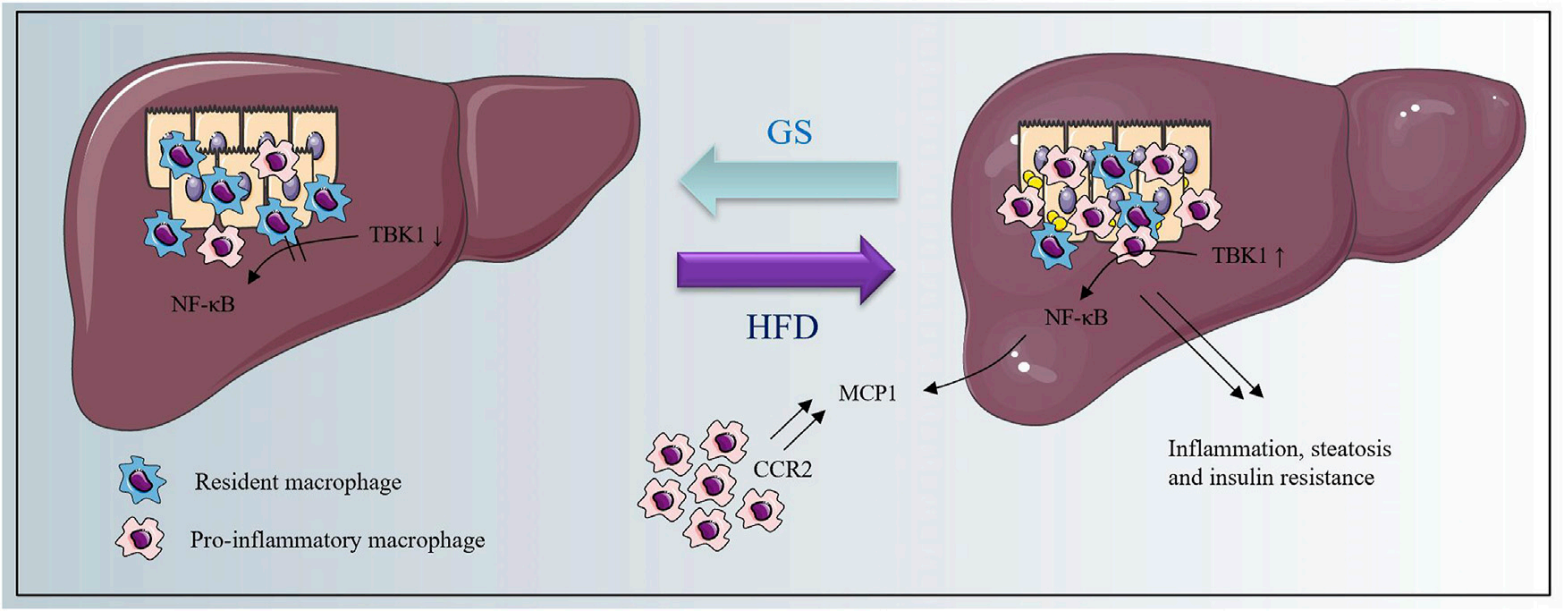
The schematic diagram.

Taken together, we found that metabolic burden would up-regulate the phosphorylation of TBK1, resulting in activation of NF-κB signaling pathway, and consequently caused an elevated expression of MCP1 to induce the macrophage recruitment and accelerate the inflammatory cascade. GS could restrain the whole process to provide therapeutic effects on metabolic dysfunction and inflammation, and the schematic diagram is shown in Figure 7.

## Discussion

NAFLD is a clinicopathological syndrome caused by intrahepatic fat accumulation, and comprises a continuum of liver pathologies ranging from steatosis to steatohepatitis, including fibrosis and cirrhosis (Eslam et al., 2020). Owing to its intimate association with obesity, NAFLD has become a progressive metabolic disease that is emerging as a global epidemic (Fan et al., 2017; Younossi, 2018). However, the complex pathogenesis of NAFLD has not yet been fully elucidated until now, and correspondingly barely pharmacological therapy was recommended in current guidelines (Aller et al., 2018).

In recent years, GS has received much attention due to its diverse pharmacological properties as an herb used in Tibetan medicine (Khobrakova et al., 2017). According to ancient Chinese medicine, GS is recognized as bitter, astringent, cold and non-toxic, and thus possesses the functions of clearing liver, purging gallbladder, eliminating heat and detoxification (Liu et al., 1995). Meanwhile, GS is attributed to lung and liver meridians, and the principal indication is jaundice with damp-heat, which means it may exert better effects on liver diseases (Yang, 1991). Based on the theory of traditional Chinese medicine, NAFLD is a syndrome of asthenia in origin and sthenia in superficiality: congenital defect, improper diet, overstrain and emotional maladjustment are all essential etiological factors, which bring about deficiency of Spleen-Qi, depression of Liver-Qi, and formation of damp-heat (Zhao and Xie, 2020; Zhou et al., 2021). Herein, the deficiency of Spleen-Qi leads to energy dysmetabolism, the depression of Liver-Qi results in impaired capacities of regulation and recovery, and the resultant pathological products and inflammatory reactions are manifested as damp-heat, which further fights with phlegm, turbidity, blood stasis and poison to promote the disease evolution (Zhou et al., 2021). Considering that stagnation of damp-heat is a common syndrome in clinical practice, we propose that GS is an effective approach for the prevention and treatment of NAFLD as its well-matched indication.

Studies have shown that GS extracted from different sorts of Gentian manifests distinct pharmacological properties, and some varieties including *Gentiana* farreri Balf. f., *Gentiana* veitchiorum Hemsl., *Gentiana* tizuensis Franch., *Gentiana* stipitata Edgew., and *Gentiana* algida Pall. are widely used in the current clinical treatment (Xu et al., 2008; Cao et al., 2014). The GS used in this study was extracted from *Gentiana* veitchiorum Hemsl. and the qualitative analysis was measured by LC-MS. Researches have revealed enormous differences in contents of active components extracted from different parts, that the largest content of ursolic acid is found to be in the flower of Gentian (Huang and Zou, 2013). Notably, our pharmacological analysis demonstrated that ursolic acid took a considerable weight in the network for GS-related targets, hence we proposed that it might be an important bioactive constituent for the curative efficacy. Considering that ursolic acid is a compound provided with anti-inflammatory and anti-oxidant properties, we detected inflammatory signaling pathways and found that GS could inhibit the phosphorylation of TBK1 and NF-κB, resulting in a declined expression of MCP1, so as to restrain the recruitment of pro-inflammatory macrophages.

The role of immune inflammation involving macrophages has been extensively investigated in NAFLD, revealing its intimate participation in multiple concurrent intrahepatic and extrahepatic events contributing to disease progression (Alharthi et al., 2020). Liver-resident Kupffer cells and hepatocytes initiate the inflammatory response which is instrumental in recruiting monocytes; meanwhile, recruited macrophages differentiate into pro-inflammatory phenotype further promoting the inflammatory cascade (Krenkel and Tacke, 2017; Kazankov et al., 2019). What’s more, macrophage activation is not restricted to the liver, but the pro-inflammatory macrophages in adipose tissues also contribute to systemically low-grade inflammation, thus boosting the evolution of NAFLD (Asghar and Sheikh, 2017; Bijnen et al., 2018). Consequently, regulating the activation and recruitment of macrophages may be an effective mean for the prevention and treatment of NAFLD (Krenkel et al., 2018).

Studies have demonstrated that TBK1 is a bi-directional regulator at the crossroads of inflammation and energy homeostasis (Zhao et al., 2018). Experimental research presented that TBK1 was highly expressed in high-fat-fed mice, which directly inhibited the expression of AMP-activated protein kinase (AMPK) to suppress respiration by reducing lipid oxidation and mitochondrial biogenesis, as well as to increase energy storage through lipogenesis and glycogenesis (Hasan et al., 2017; Chen et al., 2020). Further investigation suggested that TBK1 could inhibit the NF-κB activity by phosphorylating and inducing the degradation of IKK kinase, thus attenuating inflammatory reactions (Cai et al., 2020). As a dual inhibitor of TBK1 and IKK, AML has represented splendid curative effects that can even reverse the severity of NAFLD, and the TBK1-specific knockout in obese mice also brings about amelioration on metabolic disorders including lipid accumulation and inflammation (Reilly et al., 2013; Zhou et al., 2020). Therefore, plasmid vectors for shTBK1 and AML were used in this study to verify the mechanisms of TBK1 both *in vitro* and *in vivo*, while a co-culture system for hepatocytes and BMDMs was constructed to confirm the critical role of macrophages for inflammatory cascade.

Limitations should be acknowledged in this research, FSC and SSC was used to exclude the cell debris and doublets for gating strategy of FACS analysis, but it would be better to use viability dye to obtain more accurate and reproducible data. Meanwhile, this study is just a preliminary exploration, some Tibetan herbs have received more and more attention in recent years, such as saffron crocus and cordyceps sinensis; however, research on GS is still rare (Moshiri et al., 2015; Ong and Aziz, 2017). Our investigation has demonstrated the regulatory effects of GS on pro-inflammatory macrophages, while researches on other signaling pathways and regulators are also warranted as to its various therapeutic effects. Furthermore, LC-MS was conducted only for qualitative analysis in this current study, and gentiopicrin was identified as a control since it was the standard sample for Gentian and reported to be largely contained in GS; nevertheless, other compositions should also be detected in subsequent researches. For example, evidence reveals the content of ursolic acid in the flower of Gentian is much more than that in the rhizome, while it is just on the opposite for gentiopicrin, hence we propose that ursolic acid is an important bioactive constituent, which is consisted with our bioinformatics analysis. Consequently, researches on the mechanisms of exact pharmacodynamic components are conducive to further exploration and application of GS, which may provide new insights to improve the current standardized intervention strategy.

## Data Availability

The original contributions presented in the study are included in the article/Supplementary Material, further inquiries can be directed to the corresponding authors.

## References

[B1] Alharthi J. Latchoumanin O. George J. Eslam M. (2020). Macrophages in Metabolic Associated Fatty Liver Disease. World J. Gastroenterol. 26 (16), 1861–1878. 10.3748/wjg.v26.i16.1861 32390698 PMC7201150

[B2] Aller R. Fernández-Rodríguez C. Lo Iacono O. Bañares R. Abad J. Carrión J. A. (2018). Erratum to «Consensus Document. Management of Non-alcoholic Fatty Liver Disease (NAFLD). Clinical Practice Guideline» [Gastroenterol Hepatol. 2018;41(5):328-349]. Gastroenterol. Hepatol. 41 (5), 475–476. 10.1016/j.gastrohep.2017.12.00310.1016/j.gastrohep.2018.05.011 29631866

[B3] Asghar A. Sheikh N. (2017). Role of Immune Cells in Obesity Induced Low Grade Inflammation and Insulin Resistance. Cel. Immunol. 315, 18–26. 10.1016/j.cellimm.2017.03.001 28285710

[B4] Bijnen M. Josefs T. Cuijpers I. Maalsen C. J. van de Gaar J. Vroomen M. (2018). Adipose Tissue Macrophages Induce Hepatic Neutrophil Recruitment and Macrophage Accumulation in Mice. Gut 67 (7), 1317–1327. 10.1136/gutjnl-2016-313654 29074725

[B5] Brunt E. M. Kleiner D. E. Wilson L. A. Belt P. Neuschwander-Tetri B. A. (2011). Nonalcoholic Fatty Liver Disease (NAFLD) Activity Score and the Histopathologic Diagnosis in NAFLD: Distinct Clinicopathologic Meanings. Hepatology 53 (3), 810–820. 10.1002/hep.24127 21319198 PMC3079483

[B6] Cai H. Yan L. Liu N. Xu M. Cai H. (2020). IFI16 Promotes Cervical Cancer Progression by Upregulating PD-L1 in Immunomicroenvironment through STING-TBK1-NF-kB Pathway. Biomed. Pharmacother. 123, 109790. 10.1016/j.biopha.2019.109790 31896065

[B7] Cao H. F. Zhao Z. L. Ga W. (2014). Research Advances in Tibetan Medicine: Gentiana Scabra. J. Chin. Med. Mat. 37 (6), 1087–1093. 10.13863/j.issn1001-4454.2014.06.018

[B8] Chen Y. L. Zheng Y. Y. Dai Y. C. Zhang Y. L. Tang Z. P. (2019). Systems Pharmacology Approach Reveals Protective Mechanisms of Jian-Pi Qing-Chang Decoction on Ulcerative Colitis. World J. Gastroenterol. 25 (21), 2603–2622. 10.3748/wjg.v25.i21.2603 31210713 PMC6558442

[B9] Chen S. Liu S. Wang J. Wu Q. Wang A. Guan H. (2020). TBK1-Mediated DRP1 Targeting Confers Nucleic Acid Sensing to Reprogram Mitochondrial Dynamics and Physiology. Mol. Cel. 80 (5), 810–827.e7. 10.1016/j.molcel.2020.10.018 33171123

[B10] Eslam M. Newsome P. N. Sarin S. K. Anstee Q. M. Targher G. Romero-Gomez M. (2020). A New Definition for Metabolic Dysfunction-Associated Fatty Liver Disease: An International Expert Consensus Statement. J. Hepatol. 73 (1), 202–209. 10.1016/j.jhep.2020.03.039 32278004

[B11] Fan J. G. Kim S. U. Wong V. W. (2017). New Trends on Obesity and NAFLD in Asia. J. Hepatol. 67 (4), 862–873. 10.1016/j.jhep.2017.06.003 28642059

[B12] Fang Y. L. Chen H. Wang C. L. Liang L. (2018). Pathogenesis of Non-alcoholic Fatty Liver Disease in Children and Adolescence: From "Two Hit Theory" to "Multiple Hit Model". World J. Gastroenterol. 24 (27), 2974–2983. 10.3748/wjg.v24.i27.2974 30038464 PMC6054950

[B13] Geng Z. Li X. B. Hou Y. Liu Q. Q. Liu S. B. Tian Q. (2010). Study on Effective Components of Gentiana Veitchiorum Hemsl. Against Chronic Bronchitis in Mice. J. Chin. Med. Mat. 33 (3), 428–431. 10.13863/j.issn1001-4454.2010.03.041

[B14] Hasan M. Gonugunta V. K. Dobbs N. Ali A. Palchik G. Calvaruso M. A. (2017). Chronic Innate Immune Activation of TBK1 Suppresses mTORC1 Activity and Dysregulates Cellular Metabolism. Proc. Natl. Acad. Sci. U. S. A. 114 (4), 746–751. 10.1073/pnas.1611113114 28069950 PMC5278463

[B15] Hirsova P. Ibrahim S. H. Krishnan A. Verma V. K. Bronk S. F. Werneburg N. W. (2016). Lipid-Induced Signaling Causes Release of Inflammatory Extracellular Vesicles from Hepatocytes. Gastroenterology 150 (4), 956–967. 10.1053/j.gastro.2015.12.037 26764184 PMC4808464

[B16] Huang H. Zou S. Q. (2013). Determination of Oleanolic Acid and Ursolic Acid in Different Parts of Gentiana Tizuensis and Gentiana Farreri by RP-HPLC. Chin. J. Pharm. Anal. 33 (7), 1239–1242. 10.16155/j.0254-1793.2013.07.008

[B17] Kazankov K. Jørgensen S. M. D. Thomsen K. L. Møller H. J. Vilstrup H. George J. (2019). The Role of Macrophages in Nonalcoholic Fatty Liver Disease and Nonalcoholic Steatohepatitis. Nat. Rev. Gastroenterol. Hepatol. 16 (3), 145–159. 10.1038/s41575-018-0082-x 30482910

[B18] Khobrakova V. B. Budaeva E. R. Olennikov D. N. Zilfikarov I. N. (2017). Immunomodulating Activity of Extract of gentiana Algida Pall. Pharm. Chem. J. 51 (5), 384–387. 10.1007/s11094-017-1618-z

[B19] Krenkel O. Tacke F. (2017). Liver Macrophages in Tissue Homeostasis and Disease. Nat. Rev. Immunol. 17 (5), 306–321. 10.1038/nri.2017.11 28317925

[B20] Krenkel O. Puengel T. Govaere O. Abdallah A. T. Mossanen J. C. Kohlhepp M. (2018). Therapeutic Inhibition of Inflammatory Monocyte Recruitment Reduces Steatohepatitis and Liver Fibrosis. Hepatology 67 (4), 1270–1283. 10.1002/hep.29544 28940700

[B21] Le D. H. Le L. (2016). Systems Pharmacology: A Unified Framework for Prediction of Drug-Target Interactions. Curr. Pharm. Des. 22 (23), 3569–3575. 10.2174/1381612822666160418121534 27087598

[B22] Leroux A. Ferrere G. Godie V. Cailleux F. Renoud M. L. Gaudin F. (2012). Toxic Lipids Stored by Kupffer Cells Correlates with Their Pro-inflammatory Phenotype at an Early Stage of Steatohepatitis. J. Hepatol. 57 (1), 141–149. 10.1016/j.jhep.2012.02.028 22425624

[B23] Li P. Tang J. Li A. Hou Y. Tian Q. (2008). Therapeutic Effect of Gentiana Veitchiorum Hemsl. On Hepatic Fibrosis Induced by Dimethylnitrosamine. Lishizhen Med. Mater. Res. 19 (7), 1565–1567. 10.3969/j.issn.1008-0805.2008.07.008

[B24] Liu H. Q. Liu Y. R. Zhu Z. Q. Chen Y. R. (1995). Medicinal Plant Resources of Gentiana in Qinghai Province. J. Chin. Med. Mat. 18 (3), 119–125. 10.1006/anbo.1995.1002

[B25] Lu Y. C. Lin P. C. Wang H. (2009). Determination of Four Active Components in Gentiana Scabra by HPLC. Lishizhen Med. Mater. Res. 20 (8), 1882. 10.1002/pca.1138

[B26] Martin A. Ochagavia M. E. Rabasa L. C. Miranda J. Fernandez-de-Cossio J. Bringas R. (2010). BisoGenet: a New Tool for Gene Network Building, Visualization and Analysis. BMC Bioinformatics 11, 91. 10.1186/1471-2105-11-91 20163717 PMC3098113

[B27] Morinaga H. Mayoral R. Heinrichsdorff J. Osborn O. Franck N. Hah N. (2015). Characterization of Distinct Subpopulations of Hepatic Macrophages in HFD/obese Mice. Diabetes 64 (4), 1120–1130. 10.2337/db14-1238 25315009 PMC4375077

[B28] Moshiri M. Vahabzadeh M. Hosseinzadeh H. (2015). Clinical Applications of Saffron (Crocus Sativus) and its Constituents: A Review. Drug Res. (Stuttg) 65 (6), 287–295. 10.1055/s-0034-1375681 24848002

[B29] Obstfeld A. E. Sugaru E. Thearle M. Francisco A. M. Gayet C. Ginsberg H. N. (2010). C-C Chemokine Receptor 2 (CCR2) Regulates the Hepatic Recruitment of Myeloid Cells that Promote Obesity-Induced Hepatic Steatosis. Diabetes 59 (4), 916–925. 10.2337/db09-1403 20103702 PMC2844839

[B30] Olennikov D. N. Kashchenko N. I. Chirikova N. K. Tankhaeva L. M. (2015). Iridoids and Flavonoids of Four Siberian Gentians: Chemical Profile and Gastric Stimulatory Effect. Molecules 20 (10), 19172–19188. 10.3390/molecules201019172 26506331 PMC6331849

[B31] Ong B. Y. Aziz Z. (2017). Efficacy of Cordyceps Sinensis as an Adjunctive Treatment in Kidney Transplant Patients: A Systematic-Review and Meta-Analysis. Complement. Ther. Med. 30, 84–92. 10.1016/j.ctim.2016.12.007 28137532

[B32] Reilly S. M. Chiang S. H. Decker S. J. Chang L. Uhm M. Larsen M. J. (2013). An Inhibitor of the Protein Kinases TBK1 and IKK-ɛ Improves Obesity-Related Metabolic Dysfunctions in Mice. Nat. Med. 19 (3), 313–321. 10.1038/nm.3082 23396211 PMC3594079

[B33] Remmerie A. Scott C. L. (2018). Macrophages and Lipid Metabolism. Cell Immunol 330, 27–42. 10.1016/j.cellimm.2018.01.020 29429624 PMC6108423

[B34] Rives C. Fougerat A. Ellero-Simatos S. Loiseau N. Guillou H. Gamet-Payrastre L. (2020). Oxidative Stress in NAFLD: Role of Nutrients and Food Contaminants. Biomolecules 10 (12), 1702. 10.3390/biom10121702 PMC7767499 33371482

[B35] Ru J. Li P. Wang J. Zhou W. Li B. Huang C. (2014). TCMSP: a Database of Systems Pharmacology for Drug Discovery from Herbal Medicines. J. Cheminform. 6, 13. 10.1186/1758-2946-6-13 24735618 PMC4001360

[B36] Shannon P. Markiel A. Ozier O. Baliga N. S. Wang J. T. Ramage D. (2003). Cytoscape: a Software Environment for Integrated Models of Biomolecular Interaction Networks. Genome Res. 13 (11), 2498–2504. 10.1101/gr.1239303 14597658 PMC403769

[B37] Tang Y. Li M. Wang J. Pan Y. Wu F. X. (2015). CytoNCA: a Cytoscape Plugin for Centrality Analysis and Evaluation of Protein Interaction Networks. Biosystems 127, 67–72. 10.1016/j.biosystems.2014.11.005 25451770

[B38] Tilg H. Moschen A. R. (2010). Evolution of Inflammation in Nonalcoholic Fatty Liver Disease: the Multiple Parallel Hits Hypothesis. Hepatology 52 (5), 1836–1846. 10.1002/hep.24001 21038418

[B39] Xu C. M. Dong Q. Xing Y. X. Hu F. Z. (2008). Determination of Four Iridoid Glycosides in Tibetan Medicine Gentiana Tizuensis Franch and Gentiana Farreri by HPLC. Nat. Prod. Res. Dev. 20 (3), 466. 10.16333/j.1001-6880.2008.03.017

[B40] Yang Y. C. (1991). Tibetan Medicine Records. Xining: Qinghai People Press, 186–189.

[B41] Younossi Z. Anstee Q. M. Marietti M. Hardy T. Henry L. Eslam M. (2018). Global burden of NAFLD and NASH: Trends, Predictions, Risk Factors and Prevention. Nat. Rev. Gastroenterol. Hepatol. 15 (1), 11–20. 10.1038/nrgastro.2017.109 28930295

[B42] Younossi Z. M. (2018). Non-alcoholic Fatty Liver Disease - A Global Public Health Perspective. J. Hepatol. 70 (3), 531–544. 10.1016/j.jhep.2018.10.033 30414863

[B43] Zhang W. F. Wu Y. K. Mu D. Gong J. P. Wu C. X. Huang C. (2014). Kupffer Cells: Increasingly Significant Role in Nonalcoholic Fatty Liver Disease. Ann. Hepatol. 13 (5), 489–495. 10.1016/S1665-2681(19)31247-5 25152980

[B44] Zhao H. Xie W. (2020). Treatment of Non-alcoholic Fatty Liver Disease with Integrated Traditional Chinese Medicine. Chin. J. Clin. 48 (1), 16–18. 10.3969/j.issn.2095-8552.2020.01.006

[B45] Zhao P. Wong K. I. Sun X. Reilly S. M. Uhm M. Liao Z. (2018). TBK1 at the Crossroads of Inflammation and Energy Homeostasis in Adipose Tissue. Cell 172 (4), 731–743.e12. 10.1016/j.cell.2018.01.007 29425491 PMC5808582

[B46] Zheng Y. Huang C. Zhao L. Chen Y. Liu F. (2021). Regulation of Decorin by Ursolic Acid Protects against Non-alcoholic Steatohepatitis. Biomed. Pharmacother. 143, 112166. 10.1016/j.biopha.2021.112166 34560554

[B47] Zhou Z. Qi J. Lim C. W. Kim J. W. Kim B. (2020). Dual TBK1/IKKε Inhibitor Amlexanox Mitigates Palmitic Acid-Induced Hepatotoxicity and Lipoapoptosis *In Vitro* . Toxicology 444, 152579. 10.1016/j.tox.2020.152579 32905826

[B48] Zhou L. N. Li J. X. Xue D. Y. (2021). Advances in Traditional Chinese Medicine Research of Non-alcoholic Fatty Liver Disease. Shanghai J. Tradit. Chin. Med. 55 (3), 93–97. 10.16305/j.1007-1334.2021.2004219

