# Supplementary material for: Gentiana scabra Restrains Hepatic Pro-Inflammatory Macrophages to Ameliorate Non-Alcoholic Fatty Liver Disease
Source: Front Pharmacol. 2022 Jan 18;12:816032. doi: 10.3389/fphar.2021.816032 (PMC8803634; doi:10.3389/fphar.2021.816032)
Supplement: Supplementary file 1 [file DataSheet1.DOCX]

Supplementary Materials for Gentiana Scabra Restrains Hepatic Pro-inflammatory Macrophages to Ameliorate Non-alcoholic Fatty Liver Disease

# Supplementary Figures and Tables

## Supplementary Figures


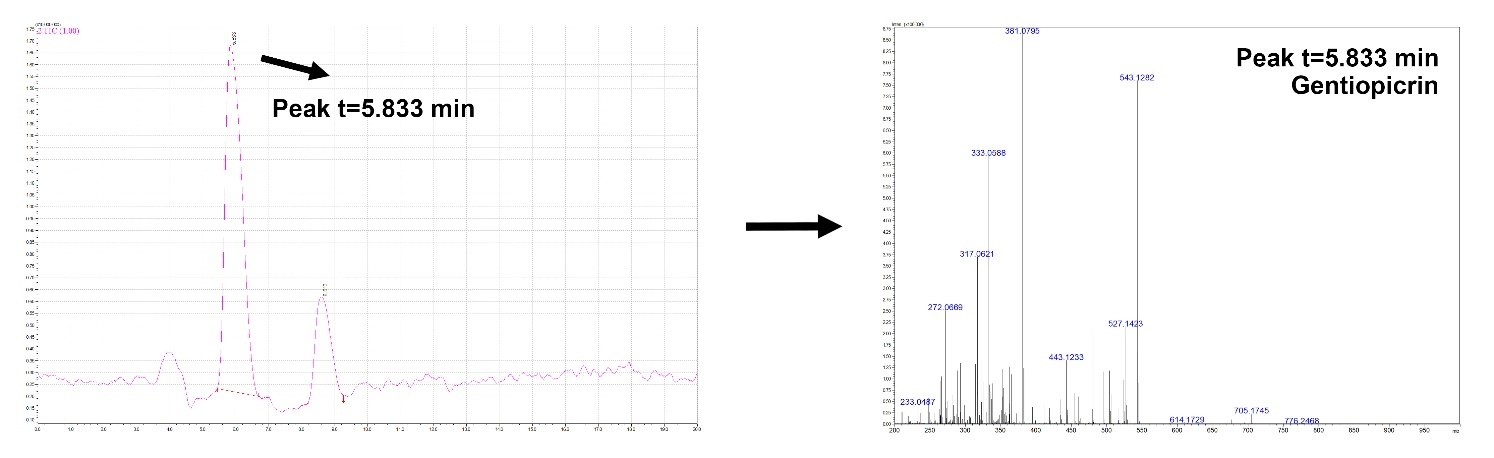


**Supplementary Figure 1.** The qualitative analysis was measured by LC-MS.


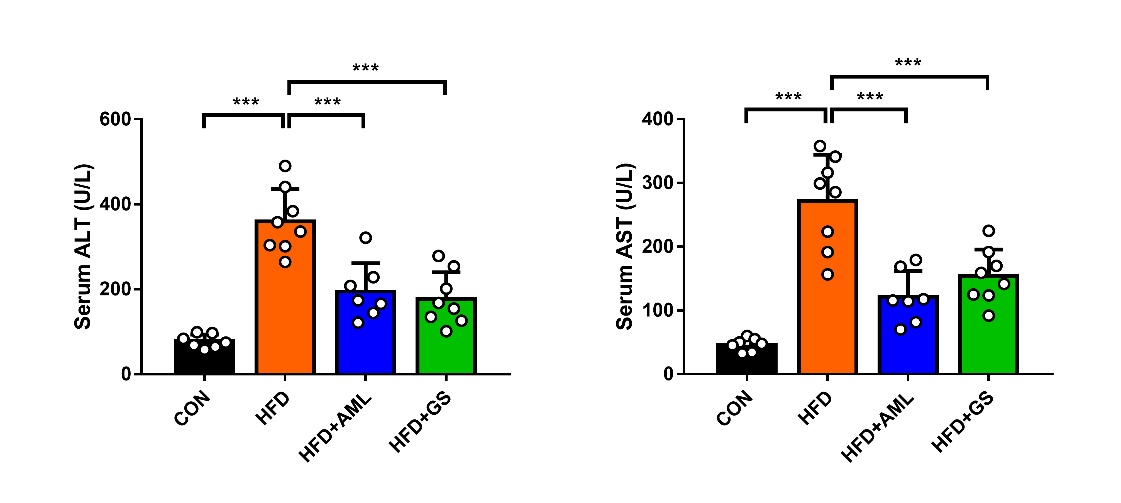


**Supplementary Figure 2.** The serum concentrations of alanine aminotransferase (ALT) and aspartate aminotransferase (AST) in high-fat-fed mice, n=7-8. Results are presented as means ± SD. **P<0.05, **P<0.01, ***P<0.001*.

## Supplementary Tables

**Supplementary Table 1.** Candidate Compounds of GS.

| NO | Chemname | Molecule ID | MW | OB | DL | Pubchem Cid |
| --- | --- | --- | --- | --- | --- | --- |
| 1 | Abscisic acid | MOL005432 | 264.35 | 63.67 | 0.13 | 5280896 |
| 2 | Mangiferin | MOL004525 | 422.37 | 13.71 | 0.75 | 5281647 |
| 3 | Gentiopicrin | MOL000646 | 356.36 | 22.98 | 0.39 | 88708 |
| 4 | Isoorientin | MOL000498 | 448.41 | 23.30 | 0.76 | 114776 |
| 5 | Oleanolic acid | MOL000263 | 456.78 | 29.02 | 0.76 | 10494 |
| 6 | Swertiajaponin | MOL003137 | 462.44 | 32.12 | 0.78 | 442659 |
| 7 | Ursolic acid | MOL000511 | 456.78 | 16.77 | 0.75 | 64945 |

**Supplementary Table 2.** Potential Targets of GS.

| NO | Candidate Compound Name | Target Protein Name | Uniprot ID | Target Gene Name |
| --- | --- | --- | --- | --- |
| 1 | Abscisic acid | Prostaglandin G/H synthase 2 | P35354 | PTGS2 |
| 2 | Abscisic acid | Gamma-aminobutyric acid receptor subunit alpha-1 | P14867 | GABRA1 |
| 3 | Abscisic acid | Nuclear receptor coactivator 2 | Q15596 | NCOA2 |
| 4 | Mangiferin | Protein-tyrosine phosphatase, non-receptor type 1 | P18031 | PTPN1 |
| 5 | Mangiferin | DNA topoisomerase II | P11388 | TOP2A |
| 6 | Gentiopicrin | NRH dehydrogenase [quinone] 2 | P16083 | NQO2 |
| 7 | Gentiopicrin | Carbonic anhydrase II | P00918 | CA2 |
| 8 | Gentiopicrin | Gamma-aminobutyric acid receptor subunit alpha-1 | P14867 | GABRA1 |
| 9 | Gentiopicrin | Ig gamma-1 chain C region | P01857 | IGHG1 |
| 10 | Gentiopicrin | Trypsin-1 | P07477 | PRSS1 |
| 11 | Isoorientin | Protein-tyrosine phosphatase, non-receptor type 1 | P18031 | PTPN1 |
| 12 | Isoorientin | Nuclear factor erythroid 2-related factor 2 | Q16236 | NFE2L2 |
| 13 | Isoorientin | NAD(P)H dehydrogenase [quinone] 1 | P15559 | NQO1 |
| 14 | Oleanolic acid | Caspase-9 | P55211 | CASP9 |
| 15 | Oleanolic acid | Caspase-3 | P42574 | CASP3 |
| 16 | Oleanolic acid | Heme oxygenase 1 | P09601 | HMOX1 |
| 17 | Oleanolic acid | Intercellular adhesion molecule 1 | P05362 | ICAM1 |
| 18 | Oleanolic acid | NAD(P)H dehydrogenase [quinone] 1 | P15559 | NQO1 |
| 19 | Oleanolic acid | Pancreatic alpha-amylase | P04746 | AMY2A |
| 20 | Swertiajaponin | DNA topoisomerase II | Q01879 | TOP2 |
| 21 | Swertiajaponin | Proto-oncogene serine/threonine-protein kinase Pim-1 | P11309 | PIM1 |
| 22 | Swertiajaponin | Protein-tyrosine phosphatase, non-receptor type 1 | P18031 | PTPN1 |
| 23 | Ursolic acid | Urokinase-type plasminogen activator | P00749 | PLAU |
| 24 | Ursolic acid | Cathepsin B | P07858 | CTSB |
| 25 | Ursolic acid | Vascular endothelial growth factor A | P15692 | VEGFA |
| 26 | Ursolic acid | Apoptosis regulator Bcl-2 | P10415 | BCL2 |
| 27 | Ursolic acid | 72 kDa type IV collagenase | P08253 | MMP2 |
| 28 | Ursolic acid | Cell division protein kinase 4 | P11802 | CDK4 |
| 29 | Ursolic acid | Tumor necrosis factor | P01375 | TNF |
| 30 | Ursolic acid | Transcription factor AP-1 | P05412 | JUN |
| 31 | Ursolic acid | Interleukin-6 | P05231 | IL6 |
| 32 | Ursolic acid | Cell division protein kinase 6 | Q00534 | CDK6 |
| 33 | Ursolic acid | Cellular tumor antigen p53 | P04637 | TP53 |
| 34 | Ursolic acid | Mitogen-activated protein kinase 8 | P45983 | MAPK8 |
| 35 | Ursolic acid | Prostaglandin G/H synthase 2 | P35354 | PTGS2 |
| 36 | Ursolic acid | Fatty acid synthase | P49327 | FASN |
| 37 | Ursolic acid | Interstitial collagenase | P03956 | MMP1 |
| 38 | Ursolic acid | Stromelysin-1 | P08254 | MMP3 |
| 39 | Ursolic acid | Heparin-binding growth factor 2 | P09038 | FGF2 |
| 40 | Ursolic acid | Stromelysin-2 | P09238 | MMP10 |
| 41 | Ursolic acid | Interleukin-1 beta | P01584 | IL1B |
| 42 | Ursolic acid | E-selectin | P16581 | SELE |
| 43 | Ursolic acid | Prostaglandin E2 receptor EP3 subtype | P43115 | PTGER3 |
| 44 | Ursolic acid | Prostaglandin G/H synthase 1 | P23219 | PTGS1 |
| 45 | Ursolic acid | Transcription factor p65 | Q04206 | RELA |
| 46 | Ursolic acid | Signal transducer and activator of transcription 3 | P40763 | STAT3 |
| 47 | Ursolic acid | G1/S-specific cyclin-D1 | P24385 | CCND1 |
| 48 | Ursolic acid | Bcl-2-like protein 1 | Q07817 | BCL2L1 |
| 49 | Ursolic acid | Proto-oncogene c-Fos | P01100 | FOS |
| 50 | Ursolic acid | Cyclin-dependent kinase inhibitor 1 | P38936 | CDKN1A |
| 51 | Ursolic acid | Apoptosis regulator BAX | Q07812 | BAX |
| 52 | Ursolic acid | Caspase-9 | P55211 | CASP9 |
| 53 | Ursolic acid | Matrix metalloproteinase-9 | P14780 | MMP9 |
| 54 | Ursolic acid | Caspase-3 | P42574 | CASP3 |
| 55 | Ursolic acid | NF-kappa-B inhibitor alpha | P25963 | NFKBIA |
| 56 | Ursolic acid | Caspase-8 | Q14790 | CASP8 |
| 57 | Ursolic acid | Intercellular adhesion molecule 1 | P05362 | ICAM1 |
| 58 | Ursolic acid | Cyclic AMP-responsive element-binding protein 1 | P16220 | CREB1 |
| 59 | Ursolic acid | Induced myeloid leukemia cell differentiation protein Mcl-1 | Q07820 | MCL1 |
| 60 | Ursolic acid | Protein kinase C gamma type | P05129 | PRKCG |
| 61 | Ursolic acid | Cyclic AMP-dependent transcription factor ATF-2 | P15336 | ATF2 |
| 62 | Ursolic acid | Granulocyte-macrophage colony-stimulating factor | P04141 | CSF2 |
| 63 | Ursolic acid | Platelet endothelial cell adhesion molecule | P16284 | PECAM1 |
| 64 | Ursolic acid | C-Jun-amino-terminal kinase-interacting protein 2 | Q13387 | MAPK8IP2 |
| 65 | Ursolic acid | Baculoviral IAP repeat-containing protein 5 | O15392 | BIRC5 |
| 66 | Ursolic acid | Tyrosine-protein phosphatase non-receptor type 6 | P29350 | PTPN6 |
| 67 | Ursolic acid | Neuromodulin | P17677 | GAP43 |
| 68 | Ursolic acid | Dual oxidase 2 | Q9NRD8 | DUOX2 |
| 69 | Ursolic acid | Nitric oxide synthase, endothelial | P29474 | NOS3 |
| 70 | Ursolic acid | Tyrosine-protein phosphatase non-receptor type 1 | P18031 | PTPN1 |
| 71 | Ursolic acid | Phosphatidylinositol-3,4,5-trisphosphate 5-phosphatase 2 | H0YFZ4 | INPPL1 |
| 72 | Ursolic acid | Lipopolysaccharide-induced tumor necrosis factor-alpha factor | Q99732 | LITAF |
| 73 | Ursolic acid | G1/S-specific cyclin-D2 | P30279 | CCND2 |
| 74 | Ursolic acid | Tumor necrosis factor ligand superfamily member 6 | P48023 | FASLG |
| 75 | Ursolic acid | Caspase-1 | P29466 | CASP1 |
| 76 | Ursolic acid | Ectonucleotide pyrophosphatase/phosphodiesterase family member 7 | Q6UWV6 | ENPP7 |

**Supplementary Table 3.** Known NAFLD-related targets.

| NO | Uniprot ID | Target Protein Name | Target Gene Name | source |
| --- | --- | --- | --- | --- |
| 1 | P01375 | Tumor Necrosis Factor | TNF | Genecards |
| 2 | P40939 | Hydroxyacyl-CoA Dehydrogenase Trifunctional Multienzyme Complex Subunit Alpha | HADHA | Genecards |
| 3 | P05231 | Interleukin 6 | IL6 | Genecards |
| 4 | P01308 | Insulin | INS | Genecards |
| 5 | P23786 | Carnitine Palmitoyltransferase 2 | CPT2 | Genecards |
| 6 | Q07869 | Peroxisome Proliferator Activated Receptor Alpha | PPARA | Genecards |
| 7 | Q13133 | Nuclear Receptor Subfamily 1 Group H Member 3 | NR1H3 | Genecards |
| 8 | P24298 | Glutamic--Pyruvic Transaminase | GPT | Genecards |
| 9 | P50416 | Carnitine Palmitoyltransferase 1A | CPT1A | Genecards |
| 10 | P11310 | Acyl-CoA Dehydrogenase Medium Chain | ACADM | Genecards |
| 11 | P49748 | Acyl-CoA Dehydrogenase Very Long Chain | ACADVL | Genecards |
| 12 | Q96RI1 | Nuclear Receptor Subfamily 1 Group H Member 4 | NR1H4 | Genecards |
| 13 | P02768 | Albumin | ALB | Genecards |
| 14 | Q9NRA2 | Solute Carrier Family 17 Member 5 | SLC17A5 | Genecards |
| 15 | Q15848 | Adiponectin, C1Q And Collagen Domain Containing | ADIPOQ | Genecards |
| 16 | Q30201 | Homeostatic Iron Regulator | HFE | Genecards |
| 17 | P06213 | Insulin Receptor | INSR | Genecards |
| 18 | P04114 | Apolipoprotein B | APOB | Genecards |
| 19 | P49327 | Fatty Acid Synthase | FASN | Genecards |
| 20 | P01584 | Interleukin 1 Beta | IL1B | Genecards |
| 21 | Q16836 | Hydroxyacyl-CoA Dehydrogenase | HADH | Genecards |
| 22 | P41235 | Hepatocyte Nuclear Factor 4 Alpha | HNF4A | Genecards |
| 23 | P19440 | Gamma-Glutamyltransferase 1 | GGT1 | Genecards |
| 24 | P37231 | Peroxisome Proliferator Activated Receptor Gamma | PPARG | Genecards |
| 25 | Q15067 | Acyl-CoA Oxidase 1 | ACOX1 | Genecards |
| 26 | P55157 | Microsomal Triglyceride Transfer Protein | MTTP | Genecards |
| 27 | P41159 | Leptin | LEP | Genecards |
| 28 | Q9UJS0 | Solute Carrier Family 25 Member 13 | SLC25A13 | Genecards |
| 29 | P05181 | Cytochrome P450 Family 2 Subfamily E Member 1 | CYP2E1 | Genecards |
| 30 | P04637 | Tumor Protein P53 | TP53 | Genecards |
| 31 | P51659 | Hydroxysteroid 17-Beta Dehydrogenase 4 | HSD17B4 | Genecards |
| 32 | P20823 | HNF1 Homeobox A | HNF1A | Genecards |
| 33 | P36956 | Sterol Regulatory Element Binding Transcription Factor 1 | SREBF1 | Genecards |
| 34 | Q04206 | RELA Proto-Oncogene, NF-KB Subunit | RELA | Genecards |
| 35 | P01137 | Transforming Growth Factor Beta 1 | TGFB1 | Genecards |
| 36 | P01009 | Serpin Family A Member 1 | SERPINA1 | Genecards |
| 37 | P01116 | KRAS Proto-Oncogene, GTPase | KRAS | Genecards |
| 38 | O43772 | Solute Carrier Family 25 Member 20 | SLC25A20 | Genecards |
| 39 | P50542 | Peroxisomal Biogenesis Factor 5 | PEX5 | Genecards |
| 40 | P55084 | Hydroxyacyl-CoA Dehydrogenase Trifunctional Multienzyme Complex Subunit Beta | HADHB | Genecards |
| 41 | P02647 | Apolipoprotein A1 | APOA1 | Genecards |
| 42 | O43933 | Peroxisomal Biogenesis Factor 1 | PEX1 | Genecards |
| 43 | P28330 | Acyl-CoA Dehydrogenase Long Chain | ACADL | Genecards |
| 44 | P02649 | Apolipoprotein E | APOE | Genecards |
| 45 | Q13085 | Acetyl-CoA Carboxylase Alpha | ACACA | Genecards |
| 46 | P28288 | ATP Binding Cassette Subfamily D Member 3 | ABCD3 | Genecards |
| 47 | P35222 | Catenin Beta 1 | CTNNB1 | Genecards |
| 48 | O95477 | ATP Binding Cassette Subfamily A Member 1 | ABCA1 | Genecards |
| 49 | P08F94 | PKHD1 Ciliary IPT Domain Containing Fibrocystin/Polyductin | PKHD1 | Genecards |
| 50 | Q9UGP8 | SEC63 Homolog, Protein Translocation Regulator | SEC63 | Genecards |
| 51 | P21439 | ATP Binding Cassette Subfamily B Member 4 | ABCB4 | Genecards |
| 52 | P02771 | Alpha Fetoprotein | AFP | Genecards |
| 53 | O60683 | Peroxisomal Biogenesis Factor 10 | PEX10 | Genecards |
| 54 | P31749 | AKT Serine/Threonine Kinase 1 | AKT1 | Genecards |
| 55 | O95342 | ATP Binding Cassette Subfamily B Member 11 | ABCB11 | Genecards |
| 56 | P40855 | Peroxisomal Biogenesis Factor 19 | PEX19 | Genecards |
| 57 | P42336 | Phosphatidylinositol-4,5-Bisphosphate 3-Kinase Catalytic Subunit Alpha | PIK3CA | Genecards |
| 58 | Q13608 | Peroxisomal Biogenesis Factor 6 | PEX6 | Genecards |
| 59 | O43520 | ATPase Phospholipid Transporting 8B1 | ATP8B1 | Genecards |
| 60 | P28328 | Peroxisomal Biogenesis Factor 2 | PEX2 | Genecards |
| 61 | Q7Z412 | Peroxisomal Biogenesis Factor 26 | PEX26 | Genecards |
| 62 | P56192 | Methionyl-TRNA Synthetase | MARS | Genecards |
| 63 | P00734 | Coagulation Factor II, Thrombin | F2 | Genecards |
| 64 | P05787 | Keratin 8 | KRT8 | Genecards |
| 65 | P35670 | ATPase Copper Transporting Beta | ATP7B | Genecards |
| 66 | O00767 | Stearoyl-CoA Desaturase | SCD | Genecards |
| 67 | P14314 | Protein Kinase C Substrate 80K-H | PRKCSH | Genecards |
| 68 | Q9Y478 | Protein Kinase AMP-Activated Non-Catalytic Subunit Beta 1 | PRKAB1 | Genecards |
| 69 | Q92968 | Peroxisomal Biogenesis Factor 13 | PEX13 | Genecards |
| 70 | P10145 | C-X-C Motif Chemokine Ligand 8 | CXCL8 | Genecards |
| 71 | P00450 | Ceruloplasmin | CP | Genecards |
| 72 | P05783 | Keratin 18 | KRT18 | Genecards |
| 73 | P16219 | Acyl-CoA Dehydrogenase Short Chain | ACADS | Genecards |
| 74 | P06858 | Lipoprotein Lipase | LPL | Genecards |
| 75 | Q9NP71 | MLX Interacting Protein Like | MLXIPL | Genecards |
| 76 | P01579 | Interferon Gamma | IFNG | Genecards |
| 77 | Q96AD5 | Patatin Like Phospholipase Domain Containing 2 | PNPLA2 | Genecards |
| 78 | P42574 | Caspase 3 | CASP3 | Genecards |
| 79 | Q16134 | Electron Transfer Flavoprotein Dehydrogenase | ETFDH | Genecards |
| 80 | Q9Y5Y5 | Peroxisomal Biogenesis Factor 16 | PEX16 | Genecards |
| 81 | O00623 | Peroxisomal Biogenesis Factor 12 | PEX12 | Genecards |
| 82 | P22301 | Interleukin 10 | IL10 | Genecards |
| 83 | Q14697 | Glucosidase II Alpha Subunit | GANAB | Genecards |
| 84 | Q9BZW4 | Transmembrane 6 Superfamily Member 2 | TM6SF2 | Genecards |
| 85 | Q9H845 | Acyl-CoA Dehydrogenase Family Member 9 | ACAD9 | Genecards |
| 86 | P38571 | Lipase A, Lysosomal Acid Type | LIPA | Genecards |
| 87 | Q16822 | Phosphoenolpyruvate Carboxykinase 2, Mitochondrial | PCK2 | Genecards |
| 88 | Q86V24 | Adiponectin Receptor 2 | ADIPOR2 | Genecards |
| 89 | P35568 | Insulin Receptor Substrate 1 | IRS1 | Genecards |
| 90 | P48357 | Leptin Receptor | LEPR | Genecards |
| 91 | P02787 | Transferrin | TF | Genecards |
| 92 | P45983 | Mitogen-Activated Protein Kinase 8 | MAPK8 | Genecards |
| 93 | O76082 | Solute Carrier Family 22 Member 5 | SLC22A5 | Genecards |
| 94 | O75381 | Peroxisomal Biogenesis Factor 14 | PEX14 | Genecards |
| 95 | P12104 | Fatty Acid Binding Protein 2 | FABP2 | Genecards |
| 96 | P56589 | Peroxisomal Biogenesis Factor 3 | PEX3 | Genecards |
| 97 | P17861 | X-Box Binding Protein 1 | XBP1 | Genecards |
| 98 | P25445 | Tumor necrosis factor receptor superfamily member 6 | FAS | OMIM |
| 99 | P08581 | Hepatocyte growth factor receptor, HGF receptor | MET | OMIM |
| 100 | P50591 | Tumor necrosis factor ligand superfamily member 10 | TNFSF10 | OMIM |
| 101 | P02656 | Apolipoprotein C-III | APOC3 | OMIM |
| 102 | P07148 | Fatty acid-binding protein, liver | FABP1 | OMIM |
| 103 | O00470 | Homeobox protein Meis1 | MEIS1 | OMIM |
| 104 | O00206 | Toll-like receptor 4 | TLR4 | OMIM |
| 105 | Q9NST1 | Acylglycerol-3-phosphate O-acyltransferase | PNPLA3 | OMIM |
| 106 | Q92903 | Phosphatidate cytidylyltransferase 1 | CDS1 | OMIM |
| 107 | Q14116 | Interleukin-18 | IL18 | OMIM |
| 108 | P98161 | Polycystin-1 | PKD1 | OMIM |
| 109 | P37231 | Peroxisome proliferator-activated receptor gamma | PPARG | TTD |
| 110 | P43220 | Glucagon-like peptide 1 receptor | GLP1R | TTD |
| 111 | Q03181 | Peroxisome proliferator-activated receptor delta | PPARD | TTD |
| 112 | P54646 | AMPK subunit alpha-2 | PRKAA2 | TTD |
